# Long Noncoding RNA XIST Promotes Resistance to Lenvatinib in Hepatocellular Carcinoma Cells via Epigenetic Inhibition of NOD2

**DOI:** 10.1155/2022/4537343

**Published:** 2022-10-18

**Authors:** Anqi Duan, Hui Li, Wenlong Yu, Yongjie Zhang, Lei Yin

**Affiliations:** ^1^The Second Department of Biliary Surgery, Eastern Hepatobiliary Surgery Hospital, Second Military Medical University, Shanghai 200438, China; ^2^Department of Dermatology, Changhai Hospital, Second Military Medical University, Shanghai 200438, China

## Abstract

*Background*. Hepatocellular carcinoma (HCC) is a severe global health issue that still lacks of effective treatments. Lenvatinib is a novel tyrosine kinase inhibitor (TKI) that has been approved for the treatment of HCC. However, drug resistance is inevitable and limits the clinical application of lenvatinib. Till now, there is still little knowledge about the mechanisms under the resistance to lenvatinib in HCC. Long noncoding RNA (lncRNA) is a group of noncoding RNAs that play essential roles in various physiological activities including the chemoresistance. In the present study, through RNA sequencing, we discovered that lncRNA XIST was upregulated in HCC cells that was insensitive to lenvatinib. Mechanically, we found that lncXIST promotes lenvatinib resistance via activation of EZH2-NOD2-ERK axis in HCC cells. Our data suggest that targeting lncXIST/EZH2/NOD2/ERK axis might be a promising strategy to enhance the efficacy of lenvatinib against HCC cells.

## 1. Introduction

Hepatocellular carcinoma (HCC) is an aggressive cancer and ranks the third leading cause of cancer-related mortality [1]. It was estimated that there are over 900,000 new HCC cases and 800,000 deaths worldwide each year [2]. Many factors such as chronic viral hepatitis type B/C, excessive alcohol consumption, and exposure to aflatoxin can contribute to the HCC. Mechanically, various growth factors like vascular growth factor (PDGF), vascular endothelial growth factor (VEGF), and fibroblast growth factor (FGF) are involved in the progression of HCC [3]. Based on that, the Food and Drug Administration (FDA) approved the sorafenib, an oral tyrosine kinase inhibitor (TKI), for the treatment of HCC [4]. However, sorafenib showed limited clinical benefits in advanced HCC clinical treatment, and the overall 5-year survival rate is relatively low [4]. Lenvatinib was the second first-line drug that has been approved by the FDA for the treatment of HCC, due its noninferior survival benefit compared to sorafenib [5]. Lenvatinib acts mainly via inhibition of the angiogenesis in various solid cancers such as HCC, lung cancer, and thyroid cancer [6]. Although lenvatinib showed promising clinical values, the mechanisms underlying lenvatinib resistance are complicated and largely unknown. Thus, further investigations on the molecular basis of LR may provide novel insights into the identification of novel molecular targets to overcome it.

Long noncoding RNA (lncRNA) are a heterogeneous group of noncoding RNAs (ncRNAs) with a transcript size of larger than 200 nt [7]. lncRNAs have been documented to play essential roles in various biological activities such as development, differentiation, and cell death. Till now, many lncRNAs have been found to regulate HCC cell response to sorafenib. For example, LINC01089 can contribute to sorafenib chemoresistance in HCC cells [8]. LINC01273 was also able to confer resistance to sorafenib in HCC cells [9]. However, there is still little knowledge about the role of lncRNA in regulating the HCC cell response to lenvatinib.

In the current study, we identified lncXIST, which was upregulated in lenvatinib-resistant HCC cells. Further investigation found that lncXIST contributes resistance to lenvatinib via epigenetic inhibition of NOD2. Our findings suggest that lncXIST may be applied as a novel predictive biomarker and therapeutic target for lenvatinib resistance in HCC cells.

## 2. Materials and Methods

### 2.1. Cell Culture and Chemicals

Human hepatocyte LO2 cells were obtained from Shanghai Aulu Biological Technology (Shanghai, China). HCC cells (Hep3B, HepG2, Huh7, and HA22T) were obtained from Shanghai Bank of Cell Culture (Shanghai, China). The lenvatinib-resistant HepG2 (HepG2/R) was created by stepwise escalation method: parental HepG2 cell was cultured with gradually increase doses of lenvatinib from 2 nM to 1 *μ*M over 8 months. LO2 cells were cultured in DMEM, and HCC cells were cultured in RPMI1640 medium supplemented with 10% fetal bovine serum (FBS) and 1% penicillin and streptomycin in humidified air with 5% CO_2_ at 37°C. All cells were authenticated by STR profiling and tested for mycoplasma contamination by Shanghai Biowing Applied Biotechnology (Shanghai, China). SCH772984 and SB202190 were obtained from Selleck Chemicals (USA). The lenvatinib was obtained from MedChemExpress (USA), and all other routine chemicals were obtained from Sigma-Aldrich (USA).

### 2.2. Cell Viability Assay

Cell viability was measured by cell counting kit-8 (CCK-8) kit from Dojindo Lab (Japan). Briefly, 2000 cells were seeded into 96-well plates in triplicate, cultured overnight. After different treatments, cell viability was measured according to the manufacturer's guide. The results were measured at 450 nm by the microplate reader (BioTek, USA).

### 2.3. Cell Death Measurement

Cells were seeded into 6-well plate at the density of 1 × 10^5^ cells/well and subjected to various treatments after adhering overnight. Apoptosis was measured by flow cytometry using the Annexin V-FITC Apoptosis Detection kit (BD Pharmingen, USA) according to the manufacturer's guide. Data was analyzed using FlowJo software.

### 2.4. RNA Interfering

Cells were seeded into 6-well plates at the density of 1 × 10^5^ cells/well and transfected using the 5 *μ*l Lipofectamine 2000 (Life Technologies, USA), which was mixed with 2 *μ*l of 20 *μ*M siRNA in 250 *μ*l of Opi-MEM (Gibco, USA). The siRNAs/antisense oligonucleotides (ASOs) were obtained from GenePharma Ltd. (Suzhou, China).

### 2.5. Plasmid Constructs and Transfection

Full length of lncXIST and of NOD2 cDNA was synthesized according to their coding sequence and cloned into the pcDNA3.1 vector. Plasmids were transfected into cells using the Lipofectamine 2000 according to the manufacturer's guide.

### 2.6. Real-Time qPCR

Total RNA was purified from the cells using the TRIzol (Life Technologies, USA) according to the manufacturer's guide. Real-time qPCR was conducted using the SYBR Green qPCR kit (Takara, Japan). The relative gene expression was calculated using the 2-*ΔΔ*Ct method and the samples were run in triplicate.

### 2.7. RNA Sequencing

Total RNA was isolated from cells as described above. RNA sequencing was conducted using an Illumina HiSeq X-ten platform at Hangzhou HiBio Technology (China). In short, sequencing libraries were constructed using TruSeq Library Prep Kit (Illumina) and were sequenced on HisSeq7500 machine (Illumina). Reads were aligned to human hg19, and gene expression values were calculated via counting the reads mapping by the edgeR software. *p* values were adjusted using the Benjamini-Hochberg method for controlling the false discovery rate. Genes with an adjusted *p* value < 0.01 and fold change > 2 were considered differentially expressed.

### 2.8. Chromatin Immunoprecipitation (ChIP) Assay

ChIP assay was conducted using the ChIP kit (Abcam, USA) according to the manufacturer's guide. Briefly, cells were treated with formaldehyde and incubated for 10 min to generate DNA-protein complexes. Cell lysates were then sonicated to generate chromatin fragments of 200-300 bp and immunoprecipitated with EZH2 or H3K27me3-specific antibodies or IgG (negative control). Precipitated chromatin DNA was analyzed by qRT-PCR.

### 2.9. RNA Immunoprecipitation (RIP) Assay

RIP was conducted to examine whether XIST could interact to bind with potential binding proteins. EZMagna RIP kit (Sigma-Aldrich) was used according to the manufacturer's guide. Briefly, cells were lysed and cellular extract was incubated with magnetic beads conjugated with antibodies that are specific against EZH2, SUZ12, or IgG for 6 h. Then, the beads were incubated with 0.1% SDS/0.5 mg/ml proteinase K for 0.5 h. The immunoprecipitated RNA was subjected to analysis by RT-PCR.

### 2.10. Western Blots

Total proteins were extracted from cells using the CHAPS lysis buffer (Beyotime, China). The quantification of protein was measured by the Bradford assay kit (Sigma). Equal amount of protein (10 *μ*g) was loaded onto 10% SDS-PAGE and transferred to PVDF membrane (Millipore). The membrane was blocked with 5% skimmed milk for 1 h at room temperature. Then, the membrane was incubated with primary antibody at 4°C overnight. The following antibodies were used: phospho-ERK (cat: 4370; dilution: 1 : 1000; Cell Signaling Technology), ERK (cat: 4696; dilution: 1 : 1000; Cell Signaling Technology), phospho-p38 (cat: 4511; dilution: 1 : 1000; Cell Signaling Technology), p38 (cat: 8690; dilution: 1 : 1000; Cell Signaling Technology), phospho-FRS2 (cat: 3864; dilution: 1 : 1000; Cell Signaling Technology), FRS2 (cat: ab183492; dilution: 1 : 1000; Abcam), NOD2 (cat: ab31488; dilution: 1 : 1000; Abcam), EZH2 (cat: ab191250; dilution: 1 : 1000; Abcam), H3K27me3 (cat: ab6002; dilution: 1 : 1000; Abcam), and GAPDH (cat: ab8245; dilution: 1 : 5000; Abcam). Secondary antibodies conjugated to horseradish peroxidase were ordered from Sigma-Aldrich (USA). The results were visualized using Tanon™ High-sig ECL Western Blotting Substrate (Tanon, China) in Tanon-4600 instrument (Tanon, China). All experiments were repeated at least three times.

### 2.11. Statistical Analysis

All experiments were independently repeated at least three times. GraphPad Prism 7.0 (GraphPad Software, La Jolla, CA, USA) was used for data processing and statistical analysis.

Results are reported as the mean ± standard deviation (SD). Differences between the means of the groups were determined using one-way ANOVA with post hoc test. *P* < 0.05 was considered significant.

## 3. Results

### 3.1. lncXIST Is Upregulated in Lenvatinib-Resistant HepG2/R Cells and Correlated with Resistance to Lenvatinib

**Figure 1:**
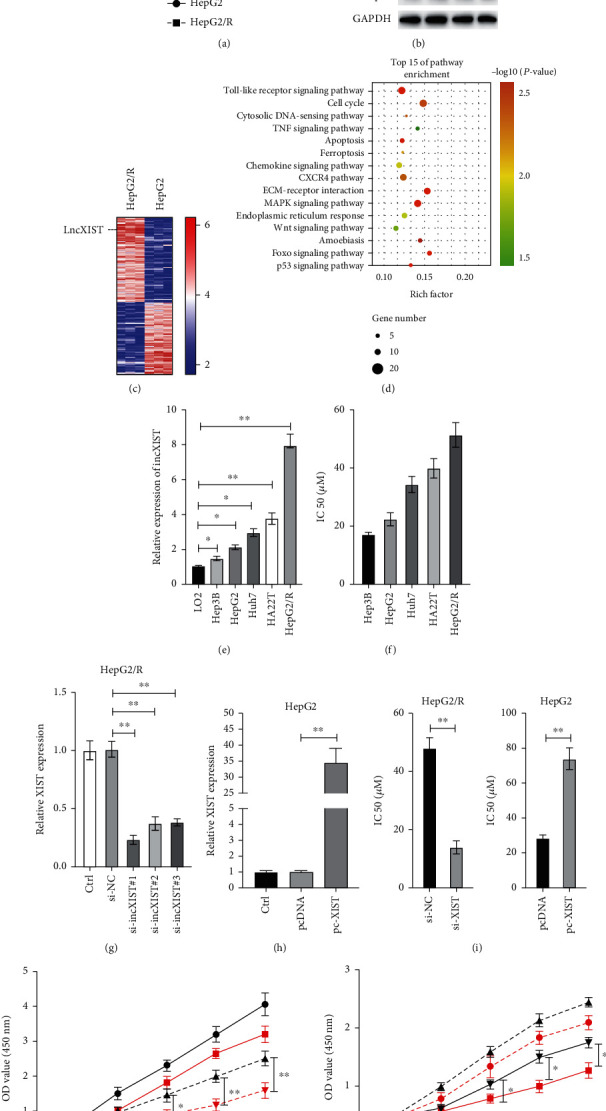
Upregulation of lncXIST confers resistance to lenvatinib in HCC cells. (a) HepG2 and HepG2/R cells were treated with indicated doses of lenvatinib for 24 h, and cellular viabilities were measured. (b) HepG2 and HepG2/R cells were treated with or without lenvatinib for 24 h, and indicated proteins were measured by western blots. (c) HepG2 and HepG2/R cells were subjected to the RNA sequencing analysis. (d) Top significantly affected pathways in HepG2/R cells based on KEGG pathway enrichment analysis. (e) Levels of lncXIST were measured in various cell lines. (f) The IC50 values of various HCC cells under the treatment of lenvatinib. (g) HepG2/R cells were transfected with various siRNAs for 24 h, and levels of lncXIST were measured. (h) HepG2 cells were transfected with empty vector (pcDNA) or lncXIST expressing vector (pc-XIST) for 24 h, and the levels of lncXIST were measured. (i) After knockdown of lncXIST or overexpressing lncXIST, the IC50 values were measured in HepG2/R and HepG2 cells, respectively. (j) HepG2/R and HepG2 cells were transfected with siRNA against lncXIST or vector expressing lncXIST, then cells were treated with or without Lenvatinib (10 *μ*M) for various time, and cellular viabilities were measured. The data was presented as mean ± SD. ^∗^*p* < 0.05 and ^∗∗^*p* < 0.01.

Firstly, we established lenvatinib-resistant HCC cells by exposing HepG2 cells to increasing doses of lenvatinib for over 9 months. As shown in Figure 1(a), lenvatinib-resistant HepG2 cells (HepG2/R) showed much higher viabilities than parental HepG2 cells after treated with various doses of lenvatinib for 24 h. HepG2 and HepG2/R cells were treated with lenvatinib (20 *μ*M) for 24 h, the FGFR signalling pathway was examined. It was found that lenvatinib successfully inhibited the phosphorylation of FRS2, a downstream FGFR signalling molecule (Figure 1(b)). In addition, lenvatinib also inhibited the phosphorylation of ERK and p38 in HepG2 cells (Figure 1(b)). At the same time, there was no significant change in the phosphorylation of FRS2, ERK, and p38 in HepG2/R cells (Figure 1(b)). In order to identify the potential lncRNAs that may contribute resistance to lenvatinib, RNA sequencing was performed. Among various lncRNAs, lncXIST was found significantly upregulated in HepG2/R cells (Figure 1(c)). Kyoto Encyclopedia of Genes and Genomes (KEGG) pathway analysis showed that several signalling pathways were highly enriched in HepG2/R cells (Figure 1(d)). Then, we measured the expression of lncXIST in various cells, and it was found that the expression of lncXIST in HCC cells was much higher than normal hepatocyte LO2 cells (Figure 1(e)). We also found that the IC50 value of lenvatinib in HCC cells is positively correlated with the expression of lncXIST (Figure 1(f)). To investigate the possible role of lncXIST in regulation HCC cell response to lenvatinib, siRNAs were applied to knockdown lncXIST. As indicated in Figure 1(g), the si-lncXIST#1 was the most efficient one, so it was used in the following experiments. We also forced the expression of lncXIST by transfecting HepG2 cells with a vector expressing lncXIST (Figure 1(h)). It was found that knockdown of lncXIST decreased HepG2/R cells IC50 to lenvatinib, while upregulation of lncXIST increased HepG2/R cells IC50 to lenvatinib (Figure 1(i)). Cell viability assays also confirmed that silencing of lncXIST markedly inhibited HepG2/R cell proliferation compared with control cells, and conversely, overexpression of lncXIST promoted proliferation of HepG2 cells with or without lenvatinib (Figure 1(j)). Taken together, those data suggest that lncXIST is upregulated in lenvatinib-resistant HepG2/R cells and contribute to resistance to lenvatinib.

### 3.2. Knockdown of lncXIST Promoted Apoptosis Induced by Lenvatinib in HCC Cells

**Figure 2:**
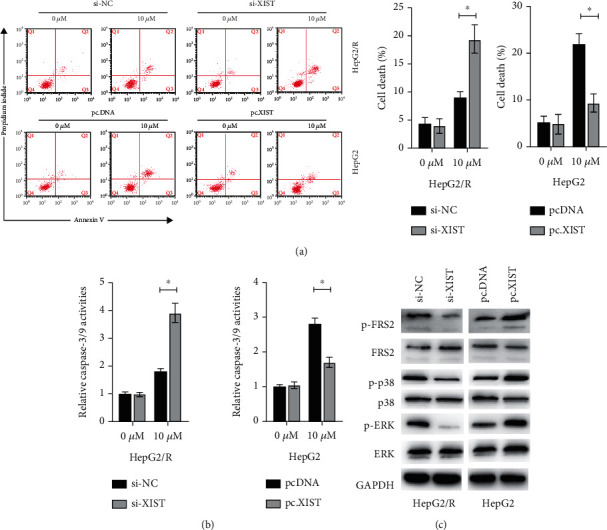
lncXIST regulates cell death induced by lenvatinib in HCC cells. (a) HCC cells were transfected with as indicated for 24 h, then cells were treated with or without lenvatinib (10 *μ*M) for another 24 h, and cell death was measured. (b) HCC cells were treated as described above, and caspase-3/7 activities were measured. (c) HepG2/R cells were transfected with si-NC or si-lncXIST and HepG2 cells were transfected with pcDNA or pc.lncXIST for 24 h, and indicated proteins were measured by western blots. The data was presented as mean ± SD. ^∗^*p* < 0.05 and ^∗∗^*p* < 0.01.

Next, we examined whether lncXIST would affect cell apoptosis. Compared with the control cells, silencing of lncXIST promoted cell death of HepG2/R cells with or without lenvatinib treatment (Figure 2(a)). At the same time, forced expression of lncXIST inhibited the cell death of HepG2 cells caused by lenvatinib (Figure 2(a)). In order to confirm the type of cell death, caspase-3/9 activity assays were conducted. It was found that silencing of lncXIST promoted the activities of caspase-3/9, while overexpression of lncXIST inhibited the activities of caspase-3/9 under the treatment of lenvatinib (Figure 2(b)). We next examined whether lncXIST regulates the FGFR signalling. As shown in Figure 2(c), silencing of lncXIST inhibited the phosphorylation of FRS2, p38, and ERK in HepG2/R cells. Meanwhile, overexpression of lncXIST increased the phosphorylation of FRS2, p38, and ERK in HepG2 cells (Figure 2(c)). Thus, lncXIST affects HCC cell sensitivity to lenvatinib via regulation of apoptosis and FGFR signalling pathway.

### 3.3. lncXIST Promotes Lenvatinib Resistance via Activation of EZH2-NOD2 Axis

**Figure 3:**
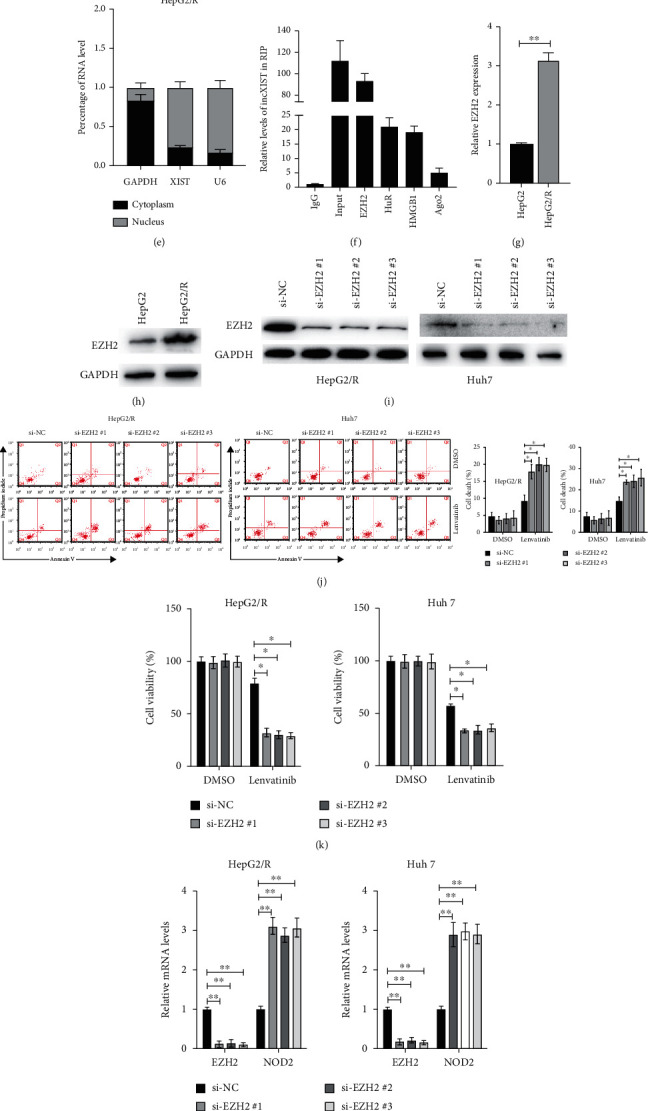
lncXIST inhibits the expression of NOD2 by binding to EZH2. (a) Heat map of differentially expressed genes in HepG2/R cells after transfection of lncXIST or control siRNAs. (b) Top significantly affected pathways in HepG2/R cells after inhibition of lncXIST based on KEGG pathway enrichment analysis. (c) Seven representative gene mRNA levels in HepG2/R cells after inhibition of lncXIST. (d) HepG2/R, HepG2, and Huh7 cells were transfected as indicated, and the protein levels of NOD2 and GAPDH were measured by western blots. (e) Subcellular distribution of lncXIST was assayed. (f) RIP assay was conducted in HepG2/R cells to show lncXIST coimmunoprecipitation with EZH2, HuR, HMGB1, and Ago2. (g) mRNA levels of EZH2 were measured by RT-PCR. (h) Protein levels of EZH2 were measured by western blots. (i) HepG2/R and Huh7 cells were transfected siRNAs against EZH2 or control siRNA for 24 h, and protein levels of EZH2 were measured. (j) HepG2/R and Huh7 cells were transfected siRNAs against EZH2 or control siRNA for 24 h, then cells were treated with or without lenvatinib (10 *μ*M) for another 24 h, and cell death was measured. (k) Cells were treated as described above, and cellular viabilities were measured. (l) HepG2/R and Huh7 cells were transfected with siRNAs against EZH2 or control siRNA for 24 h, and EZH2 and NOD2 mRNA levels were measured. (m) HepG2/R and Huh7 cells were transfected with siRNAs against EZH2 or control siRNA for 24 h, and indicated proteins were measured by western blots. (n) ChIP-qPCR assay showing EZH2 binding with the promoter region of NOD2 can be attenuated by knockdown of lncXIST. The data was presented as mean ± SD. ^∗^*p* < 0.05 and ^∗∗^*p* < 0.01.

In order to further investigate the mechanisms underlying the resistance to lenvatinib conferred by lncXIST, RNA sequencing was conducted. As indicated in Figures 3(a) and 3(b), various genes and signalling pathways were affected after knockdown of lncXIST in HepG2/R cells. Among them, NOD2 got our attention due to the highest-fold upregulation after knockdown of lncXIST which was further confirmed by RT-PCR (Figure 3(c)). Western blots also confirmed that knockdown of lncXIST led to upregulation of NOD2, while overexpression of lncXIST inhibited the NOD2 (Figure 3(d)). Additionally, we examined the distribution of lncXIST using subcellular fractionation analyses. qRT-PCR results showed that lncXIST is mainly distributed in the nucleus (Figure 3(e)), indicating that lncXIST may involve in the regulation of transcription. Amounting evidence suggests that lncRNAs can regulate the expression of genes via interaction with RNA binding proteins such as HuR, HMGB1, and EZH2 [10]. To investigate whether lncXIST could interact with those RNA-binding proteins, RNA immunoprecipitation (RIP) assays were conducted. It was revealed that lncXIST can bind with HuR, EZH2, and HMGB1. However, lncXIST interaction with EZH2 was stronger, suggesting that lncXIST interacted specifically with EZH2 in HepG2/R cells (Figure 3(f)). To further examine the correlation between lncXIST and EZH2, we measured the expression of EZH2 after silencing/overexpression of lncXIST. Surprisingly, knockdown or overexpression of lncXIST did not affect the EZH2 at both mRNA and protein levels (data not shown). Since EZH2 is a core subunit of polycomb repressive complex 2 (PRC2), it plays an essential role in regulating cancer cell response to drugs [11]. To study the role of EZH2 in HCC cell response to lenvatinib, the levels of EZH2 were examined. It was found that both the mRNA and protein levels of EZH2 were upregulated in HepG2/R cells compared to HepG2 cells (Figures 3(g) and 3(h)). Then, three siRNAs against EZH2 were applied to knockdown EZH2 in HepG2/R and Huh7 cells (Figure 3(i)). It was found that silencing of EZH2 increased the cell death induced by lenvatinib in both HepG2/R and Huh7 cells (Figure 3(j)). At the same time, cell viabilities showed that silencing of EZH2 decreased the cell viabilities of HepG2/R and Huh7 cells under the treatment of lenvatinib (Figure 3(k)). Next, we investigated the correlation between the EZH2 and NOD2. RT-PCR and western blots showed that downregulation of EZH2 led to the upregulation of NOD2 in HepG2/R and Huh7 cells (Figures 3(l) and 3(m)). Noteworthy, silencing of EZH2 also led to the inhibition of H3K27me3 (Figure 3(m)). Furthermore, chromatin immunoprecipitation (ChIP) was conducted, and it was found that knockdown of lncXIST attenuated the binding of EZH2 and H3K27 trimethylation levels across the promoter region of NOD2 (Figure 3(n)). These results suggest that lncXIST affects HCC cell response to lenvatinib, at least partly, via the epigenetic inhibition of NOD2 via interacting with EZH2 in HepG2/R cells.

### 3.4. Overexpression of NOD2 Restores Lenvatinib-Insensitive HCC Cell Response to Lenvatinib

**Figure 4:**
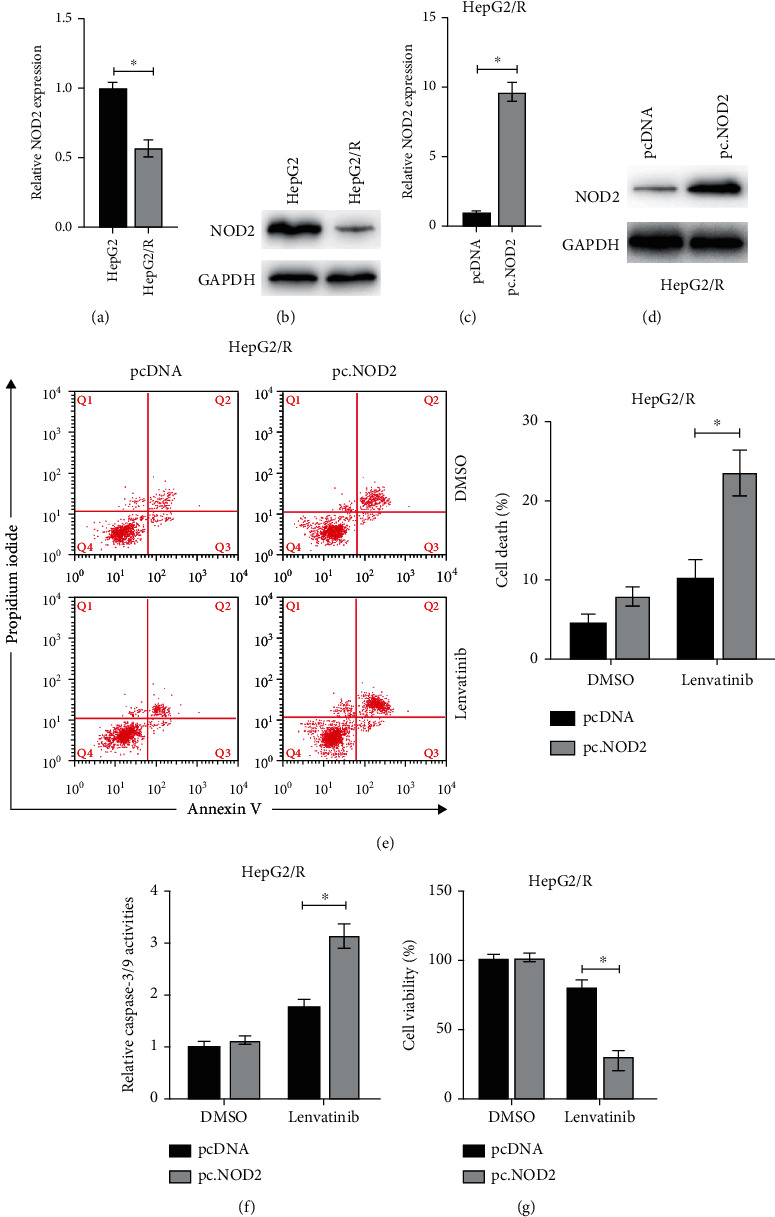
Upregulation of NOD2 can overcome resistance to lenvatinib in HCC cells. (a) mRNA levels of NOD2 were measured in HepG2 and HepG2/R cells. (b) Protein levels of NOD2 were measured in HepG2 and HepG2/R cells. (c) HepG2/R cells were transfected with pcDNA or pcNOD2 for 24 h, and mRNA levels of NOD2 were measured. (d) Protein levels of NOD2 were measured. (e) HepG2/R cells were transfected with pcDNA or pcNOD2 for 24 h, then cells were treated with or without lenvatinib (10 *μ*M) for another 24 h, and cellular death was measured. (f) Cells were treated as described above, and relative caspase-3/7 activities were measured. (g) HepG2/R cells were transfected with pcDNA or pcNOD2 for 24 h, then cells were treated with or without lenvatinib (10 *μ*M) for another 24 h, and cellular viabilities were measured. The data was presented as mean ± SD. ^∗^*p* < 0.05.

Next, we investigated the role of NOD2 in regulating HCC cell response to lenvatinib. RT-PCR and western blots showed that both the mRNA and protein levels of NOD2 were lower in HepG2/R cells compared to HepG2 cells (Figures 4(a) and 4(b)). To further analyze the function of NOD2, we overexpressed the NOD2 in HepG2/R (Figures 4(c) and 4(d)). It was found that forced expression of NOD2 increased the cell death of HepG2/R cells under the treatment of lenvatinib (Figure 4(e)). Caspases' activities also confirmed that upregulation of NOD2 resulted in increased caspases activities in HepG2/R cells under the treatment of lenvatinib (Figure 4(f)). Moreover, cellular viability assay also indicated that forced expression of NOD2 decreased viabilities of HepG2/R cells under the treatment of lenvatinib compared to the control group (Figure 4(g)). Taken together, those data indicate that forced expression of NOD2 restored lenvatinib-resistant HepG2/R cell sensitivity to lenvatinib.

### 3.5. lncXIST Contributes to Acquired Resistance to Lenvatinib Which Is Partly Relied on the Regulation of NOD2

**Figure 5:**
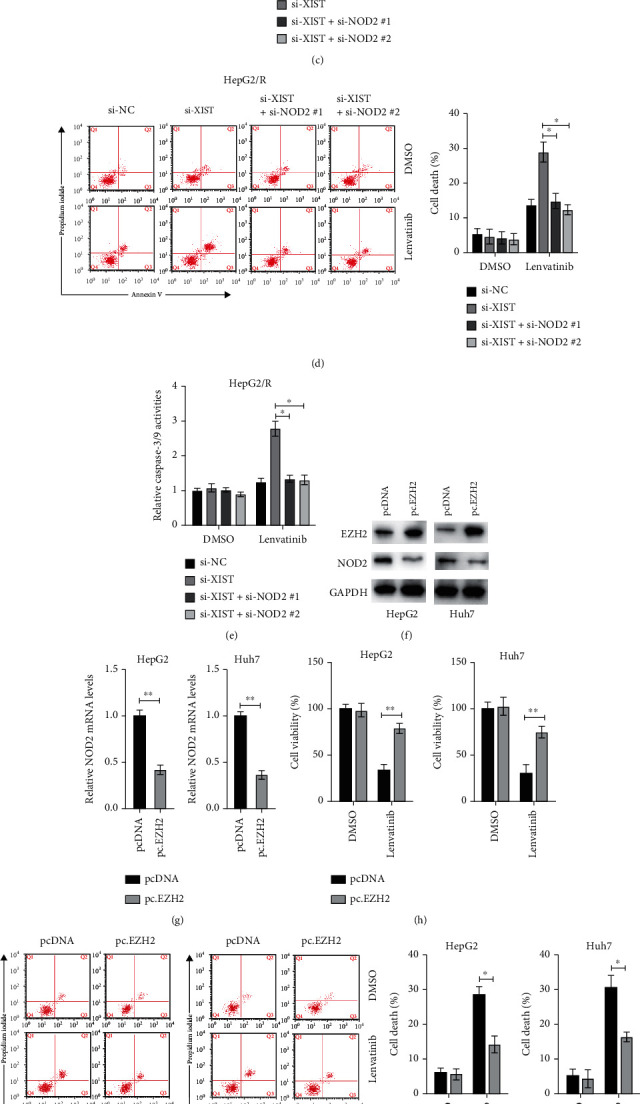
Silencing of NOD2 abrogates the effects of inhibition of lncXIST on sensitivity to lenvatinib in HCC cells. (a) HepG2/R cells were transfected with siRNAs as indicated for 24 h, and mRNA levels of NOD2 were measured. (b) HepG2/R cells were transfected with siRNAs as indicated for 24 h, and protein levels of NOD2 were measured. (c) HepG2/R cells were transfected with siRNAs as indicated for 24 h, then cells were treated with or without lenvatinib (10 *μ*M) for 24 h, and cellular viabilities were measured. (d) HepG2 cells were treated as described above, and cellular death was measured. (f) HepG2 and Huh7 cells were transfected as indicated for 24 h, and indicated proteins were measured by western blots. (g) mRNA levels of NOD2 were measured. (h) HepG2 and Huh7 cells were transfected as indicated for 24 h, then cells were treated with or without lenvatinib for another 24 h, and cellular viabilities were measured. (i) HepG2 and Huh7 cells were transfected as indicated for 24 h, then cells were treated with or without lenvatinib for another 24 h, and cellular death was measured. (j) Caspase activities were measured. The data was presented as mean ± SD; ^∗^*p* < 0.05 and ^∗∗^*p* < 0.01.

Next, we examined whether inhibition of NOD2 expression could affect the effects of knockdown of lncXIST on regulating HCC cell sensitivity to lenvatinib. As shown in Figures 5(a) and 5(b), although knockdown of lncXIST significantly increased NOD2 mRNA and protein levels, these effects were reversed by coexpression of two siRNAs against NOD2. It was found that knockdown of NOD2 reversed the effects of silencing of lncXIST on the cellular viabilities of HepG2/R cells under the treatment of lenvatinib (Figure 5(c)). Flow cytometry analysis also indicated that silencing of NOD2 reversed the effects of silencing of lncXIST on cell death induced by lenvatinib in HepG2/R cells (Figure 5(d)). Furthermore, caspases' activities also confirmed that the augmented caspase-3/9 activities caused by silencing of lncXIST under the treatment of lenvatinib could be reversed by knockdown of NOD2 in HepG2/R cells (Figure 5(e)). To further confirm the connective role of EZH2 between XIST and NOD2, we overexpressed EZH2 in HepG2 and Huh7 cells (Figure 5(f)). It was observed that overexpression of EZH2 led to the downregulation of NOD2 in both cells (Figures 5(f) and 5(g)). Overexpression of EZH2 also promoted the cellular viabilities of both HepG2 and Huh7 cells under the treatment of lenvatinib (Figure 5(h)). Moreover, overexpression of EZH2 also reduced the cellular death and activation of caspase-3/9 induced by lenvatinib in HepG2 and Huh7 cells (Figures 5(i) and 5(j)). Collectively, those findings indicate that lncXIST-EZH2-NOD2 axis confers resistance to lenvatinib in HCC cells.

### 3.6. Inhibition of NOD2 Caused Activation of ERK Which Confers Resistance to Lenvatinib

**Figure 6:**
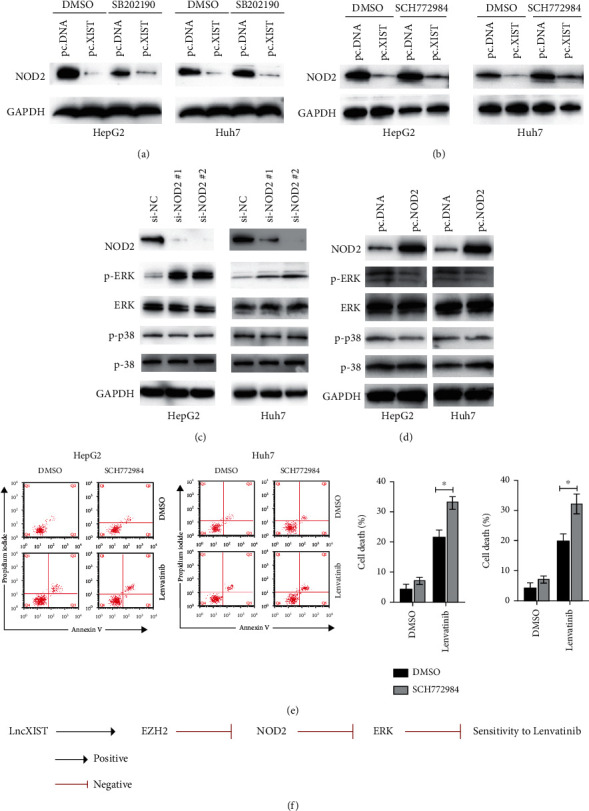
Downregulation of NOD2 caused activation of ERK that confers resistance to lenvatinib. (a) HepG2 and Huh7 cells were transfected as indicated for 24 h, then cells were treated with DMSO or SB202190 (20 *μ*M) for another 24 h, and protein levels of NOD2 were measured. (b) HepG2 and Huh7 cells were transfected as indicated for 24 h, then cells were treated with DMSO or SCH772984 (20 *μ*M) for another 24 h, and protein levels of NOD2 were measured. (c) HepG2 and Huh7 cells were transfected as indicated for 24 h, and indicated proteins were measured by western blots. (d) HepG2 and Huh7 cells were transfected as indicated for 24 h, and indicated proteins were measured by western blots. (e) HepG2 and Huh7 cells were treated with lenvatinib alone or in combination with SCH772984 (20 *μ*M) for 24 h, and cellular death was measured. (f) Proposed model by which lncXIST regulates HCC cell response to lenvatinib.

To investigate the correlation between NOD2 and MAPK signalling, p38 inhibitor (SB202190) and ERK inhibitor (SCH772984) were applied. As shown in Figures 6(a) and 6(b), both inhibitors did not affect the downregulation of NOD2 caused by overexpression of lncXIST. Therefore, we hypothesized that NOD2 might act upstream of MAPKs. Interestingly, it was found that downregulation of NOD2 led to the activation of ERK but not p38 in HepG2 and Huh7 cells (Figure 6(c)). Meanwhile, overexpression of NOD2 reduced the levels of phosphor-ERK in HepG2 and Huh7 cells (Figure 6(d)). To confirm the role of ERK in regulating cell response to lenvatinib, SCH772984 was applied. Interestingly, SCH772984 treatment increased the cell death induced by lenvatinib in both HepG2 and Huh7 cells (Figure 6(e)). Thus, downregulation of NOD2 led to the activation of ERK that might be responsible for the resistance to lenvatinib in liver cancer cells.

## 4. Discussion

HCC is one of the leading causes of cancer-related death. Lenvatinib is a multitargeted TKI that has been approved for the treatment of unresectable HCC. However, the overall response rate was only around 40% in HCC patients who received lenvatinib [12]. The clinical application of lenvatinib is often limited by drug resistance; therefore, it is necessary to unveil the mechanisms underlying the chemoresistance to lenvatinib. In the current study, we revealed that lncXIST confers resistance to lenvatinib in HCC cells. Mechanically, lncXIST can interact with EZH2 to repress the expression of NOD2.

lncRNAs, a class of noncoding RNAs, widely exist in mammalian genomes and can be detected in the tissues, body fluids, and exosomes [13]. Amounting evidence suggests that lncRNAs play essential roles in various biological activities such as cell growth, differentiation, and cell death [14]. Various lncRNAs have also been identified as a regulator of HCC cell response to TKIs. For instance, upregulation of lncNIFK-AS1 conferred resistance to sorafenib in HCC cells [15]. LncMT1JP was able to promote lenvatinib resistance in HCC cells via inhibiting apoptosis [16]. lncXIST was identified as an oncogene in various cancers including the HCC. Liu and Xu reported that lncXIST promotes progression of HCC via sponging miR-200b-3p [17]. Dong et al. found that lncXIST can accelerate the growth of HCC cells via inhibiting miR-488 [18]. lncXIST has also been documented to affect cancer cell response to chemotherapy agents. Upregulation of lncXIST confers resistance to 5-FU and doxorubicin in colorectal cancer cells [19, 20]. In the present study, we revealed for the first time that lncXIST also confers resistance to lenvatinib in HCC cells.

Previous studies have shown that lncRNAs regulate cancer cells sensitive to chemotherapeutics via various mechanisms. For instance, lncMT1JP promotes resistance to lenvatinib via acting as a competing endogenous RNA to miR-24-3p in HCC cells [16]. lncRNAs can also regulate gene transcription by recruiting histone modification enzymes or interacting with transcription factors. Chen et al. found that lncRNA CASC9 promoted resistance to gefitinib in NSCLC cells via epigenetic inhibition of DUSP1 [21]. In the current study, we revealed that lncXIST was able to bind with histone modification enzyme, EZH2, to inhibit the expression of NOD2. EZH2 is the core subunit of the PRC2 complex, which negatively regulate the gene expression via trimethylating of H3K27 [22]. It has been reported that EZH2 is overexpressed in HCC and is correlated with poor prognosis [23]. Previous studies found that EZH2 is able to regulate HCC cell sensitivity to sorafenib. For example, inhibition of EZH2 augmented the antitumor effects of sorafenib in HCC cells [24]. Interestingly, various studies found that EZH2 could interact with lncRNAs. Zhang et al. reported that lncUPK1A-AS1 was able to interact with EZH2 and promotes the proliferation of HCC cells [25]. In the current study, we found that knockdown of EZH2 could partially reversed lenvatinib resistance and promoted cell death of HCC cells. Our findings are in accordance with previous studies indicating that EZH2 could be used as a target to overcome lenvatinib resistance in HCC cells.

We also conducted RNA sequencing to find the target genes of lncXIST, and NOD2 was identified. NOD2 is one of the pivotal innate immune sensors, which can recognize pathogen infection and induce subsequent innate immune response [26]. NOD2 acts as a tumor suppressor and was found to protect mice from inflammation and obesity-dependent HCC [27]. NOD2 has also been reported to inhibit tumorigenesis and increase chemosensitivity of HCC cells via targeting AMPK pathway [28]. In line with those studies, we also found that upregulation of NOD2 prompted cell death induced by lenvatinib in HCC cells. Therefore, the levels of NOD2 might be applied as a marker to predict the lenvatinib response in HCC. Noteworthy, another study found that NOD2 was upregulated and activated in HCC tissues, and high expression of NOD2 was correlated with poor prognosis in HCC patients [29]. This discrepancy reveals the complex role of NOD2 in regulating the tumorigenesis of HCC, and more investigations are required. We also revealed that inhibition of NOD2 led to the activation of ERK in liver cancer cells. Similar to our findings, ERK activation was found enhanced in *NOD*2-/- macrophages [30]. Moreover, ERK signalling was also found upregulated in *NOD2*-/- mice [31]. In addition, our data also indicated that activation of ERK was correlated with resistance to lenvatinib. This finding is in accordance with a previous study which also showed that ERK signalling conferred resistance to lenvatinib in liver carcinoma cells [32]. Hence, targeting ERK signalling might be a strategy to overcome lenvatinib resistance.

Till now, there are many possible strategies to target the lncXIST/EZH2/NOD2/ERK axis. Many specific inhibitors against EZH2 have been developed and studied in the preclinical setting. For example, three EZH2 inhibitors, tazemetostat (EPZ-6438), GSK2816126, and CPI-1205, have moved into phase I/phase II clinical trials in patients with non-Hodgkin lymphoma and genetically defined solid tumors [33]. Clinical data showed that those EZH2 inhibitors are relatively safe. Noteworthy, another study found that EZH2 inhibitors prevented emergence of acquired resistance and augmented chemotherapeutic efficacy in both chemosensitive and chemoresistant models of small cell lung cancer [34]. In addition, SB 9200 is a novel, first-in-class oral modulator of innate immunity that is believed to act via the activation of the NOD2 pathways [35]. Although SB 9200 has a broad-spectrum antiviral activity, whether it possesses antitumor activities has not been reported yet. Amounting evidence suggests that angiogenesis and signalling through the ERK have been reported to play essential roles in hepatocarcinogenesis [36]. Sorafenib is an ERK inhibitor that has been approved for the treatment of liver carcinoma. Hence, it would be interesting to test whether those agents could enhance the efficacy of lenvatinib in liver carcinoma cells.

There are some limitations of our study. Firstly, our studies are conducted using *in vitro* assays. It would be interesting to validate our findings *in vivo*. Secondly, there might be other genes that were affected by the lncXIST/EZH2 and affect HCC cell response to lenvatinib; it is worthy for further investigations.

## 5. Conclusion

In this study, we found that lncXIST promotes resistance to lenvatinib in HCC cells. Mechanically, lncXIST interacts with EZH2 to inhibit the expression of NOD2. Overexpression of NOD2 silencing could reverse the effects of inhibition of lncXIST on HCC cell sensitivity to lenvatinib. Our findings provide novel insights into the lncXIST/EZH2/NOD2/ERK axis in regulating HCC lenvatinib resistance (Figure 6(f)).

## Data Availability

Data are available upon reasonable request to correspondence.

## References

[1] Han Z. G. (2012). Functional genomic studies: insights into the pathogenesis of liver cancer. *Annual Review of Genomics and Human Genetics*.

[2] Sung H., Ferlay J., Siegel R. L. (2021). GLOBOCAN estimates of incidence and mortality worldwide for 36 cancers in 185 countries. *CA: a Cancer Journal for Clinicians*.

[3] Semela D., Dufour J. F. (2004). Angiogenesis and hepatocellular carcinoma. *Journal of Hepatology*.

[4] Llovet J. M., Ricci S., Mazzaferro V. (2008). Sorafenib in advanced hepatocellular carcinoma. *The New England Journal of Medicine*.

[5] Kudo M., Finn R. S., Qin S. (2018). Lenvatinib versus sorafenib in first-line treatment of patients with unresectable hepatocellular carcinoma: a randomised phase 3 non-inferiority trial. *Lancet*.

[6] Tohyama O., Matsui J., Kodama K. (2014). Antitumor activity of lenvatinib (E7080): an angiogenesis inhibitor that targets multiple receptor tyrosine kinases in preclinical human thyroid cancer models. *Journal of Thyroid Research*.

[7] Heydarnezhad Asl M., Pasban Khelejani F., Bahojb Mahdavi S. Z., Emrahi L., Jebelli A., Mokhtarzadeh A. (2022). The various regulatory functions of long noncoding RNAs in apoptosis, cell cycle, and cellular senescence. *Journal of Cellular Biochemistry*.

[8] Sun J., Zheng X., Wang B. (2022). An Argonaute from exhibits endonuclease activity mediated by 5' chemically modified DNA guides. *Acta Biochim Biophys Sin (Shanghai)*.

[9] Kong H., Sun J., Zhang W., Zhang H., Li H. (2022). Long intergenic non-protein coding RNA 1273 confers sorafenib resistance in hepatocellular carcinoma via regulation of methyltransferase 3. *Bioengineered*.

[10] Ferre F., Colantoni A., Helmer-Citterich M. (2016). Revealing protein-lncRNA interaction. *Briefings in Bioinformatics*.

[11] Crea F., Paolicchi E., Marquez V. E., Danesi R. (2012). Polycomb genes and cancer: time for clinical application?. *Critical Reviews in Oncology/Hematology*.

[12] Hiraoka A., Kumada T., Kariyama K. (2019). Therapeutic potential of lenvatinib for unresectable hepatocellular carcinoma in clinical practice: multicenter analysis. *Hepatology Research*.

[13] Corrado C., Barreca M. M., Zichittella C., Alessandro R., Conigliaro A. (2021). Molecular mediators of RNA loading into extracellular vesicles. *Cell*.

[14] Adnane S., Marino A., Leucci E. (2022). LncRNAs in human cancers: signal from noise. *Trends in Cell Biology*.

[15] Chen Y. T., Xiang D., Zhao X. Y., Chu X. Y. (2021). Upregulation of lncRNA NIFK-AS1 in hepatocellular carcinoma by m6A methylation promotes disease progression and sorafenib resistance. *Human Cell*.

[16] Yu T., Yu J., Lu L. (2021). MT1JP-mediated miR-24-3p/BCL2L2 axis promotes lenvatinib resistance in hepatocellular carcinoma cells by inhibiting apoptosis. *Cellular Oncology (Dordrecht)*.

[17] Liu W. G., Xu Q. (2019). Long non-coding RNA XIST promotes hepatocellular carcinoma progression by sponging miR-200b-3p. *European Review for Medical and Pharmacological Sciences*.

[18] Dong Z., Yang J., Zheng F., Zhang Y. (2020). The expression of lncRNA XIST in hepatocellular carcinoma cells and its effect on biological function. *Journal of BUON*.

[19] Xiao Y., Yurievich U. A., Yosypovych S. V. (2017). Long noncoding RNA XIST is a prognostic factor in colorectal cancer and inhibits 5-fluorouracil-induced cell cytotoxicity through promoting thymidylate synthase expression. *Oncotarget*.

[20] Zhu J., Zhang R., Yang D. (2018). Knockdown of long non-coding RNA XIST inhibited doxorubicin resistance in colorectal cancer by upregulation of miR-124 and downregulation of SGK1. *Cellular Physiology and Biochemistry*.

[21] Chen Z., Chen Q., Cheng Z. (2020). Long non-coding RNA CASC9 promotes gefitinib resistance in NSCLC by epigenetic repression of DUSP1. *Cell Death & Disease*.

[22] Liu K. L., Zhu K., Zhang H. (2022). An overview of the development of EED inhibitors to disable the PRC2 function. *RSC Med Chem*.

[23] Bae A. N., Jung S. J., Lee J. H., Lee H., Park S. G. (2022). Clinical value of EZH2 in hepatocellular carcinoma and its potential for target therapy. *Medicina (Kaunas, Lithuania)*.

[24] Kusakabe Y., Chiba T., Oshima M. (2021). EZH1/2 inhibition augments the anti-tumor effects of sorafenib in hepatocellular carcinoma. *Scientific Reports*.

[25] Zhang D. Y., Sun Q. C., Zou X. J. (2020). Long noncoding RNA UPK1A-AS1 indicates poor prognosis of hepatocellular carcinoma and promotes cell proliferation through interaction with EZH2. *Journal of Experimental & Clinical Cancer Research*.

[26] Hruz P., Zinkernagel A. S., Jenikova G. (2009). NOD2 contributes to cutaneous defense against Staphylococcus aureus through alpha-toxin-dependent innate immune activation. *Proceedings of the National Academy of Sciences of the United States of America*.

[27] Gurses S. A., Banskar S., Stewart C., Trimoski B., Dziarski R., Gupta D. (2020). Nod2 protects mice from inflammation and obesity-dependent liver cancer. *Scientific Reports*.

[28] Ma X., Qiu Y., Sun Y. (2020). NOD2 inhibits tumorigenesis and increases chemosensitivity of hepatocellular carcinoma by targeting AMPK pathway. *Cell Death & Disease*.

[29] Zhou Y., Hu L., Tang W. (2021). Hepatic NOD2 promotes hepatocarcinogenesis via a RIP2-mediated proinflammatory response and a novel nuclear autophagy-mediated DNA damage mechanism. *Journal of Hematology & Oncology*.

[30] Anand P. K., Tait S. W., Lamkanfi M. (2011). TLR2 and RIP2 pathways mediate autophagy of Listeria monocytogenes via extracellular signal-regulated kinase (ERK) activation. *The Journal of Biological Chemistry*.

[31] Du P., Fan B., Han H. (2013). NOD2 promotes renal injury by exacerbating inflammation and podocyte insulin resistance in diabetic nephropathy. *Kidney International*.

[32] Zhao Z., Zhang D., Wu F. (2021). Sophoridine suppresses lenvatinib-resistant hepatocellular carcinoma growth by inhibiting RAS/MEK/ERK axis via decreasing VEGFR2 expression. *Journal of Cellular and Molecular Medicine*.

[33] Gulati N., Beguelin W., Giulino-Roth L. (2018). Enhancer of zeste homolog 2 (EZH2) inhibitors. *Leukemia & Lymphoma*.

[34] Gardner E. E., Lok B. H., Schneeberger V. E. (2017). Chemosensitive relapse in small cell lung cancer proceeds through an EZH2-SLFN11 axis. *Cancer Cell*.

[35] Jones M., Cunningham M. E., Wing P. (2017). SB 9200, a novel agonist of innate immunity, shows potent antiviral activity against resistant HCV variants. *Journal of Medical Virology*.

[36] Woo H. Y., Heo J. (2012). Sorafenib in liver cancer. *Expert Opinion on Pharmacotherapy*.

